# Oral Microbially-Induced Small Extracellular Vesicles Cross the Blood–Brain Barrier

**DOI:** 10.3390/ijms25084509

**Published:** 2024-04-20

**Authors:** Mahmoud Elashiry, Angelica Carroll, Jessie Yuan, Yutao Liu, Mark Hamrick, Christopher W. Cutler, Qin Wang, Ranya Elsayed

**Affiliations:** 1 Department of Periodontics, Dental College of Georgia, Augusta University, Augusta, GA 30912, USA; melashiry@augusta.edu (M.E.); ancarroll@augusta.edu (A.C.); jeyuan@augusta.edu (J.Y.); chcutler@augusta.edu (C.W.C.); 2 Department of Cellular Biology and Anatomy, Medical College of Georgia, Augusta University, Augusta, GA 30912, USA; yutliu@augusta.edu (Y.L.); mhamrick@augusta.edu (M.H.); 3 Department of Neuroscience and Regenerative Medicine, Medical College of Georgia, Augusta University, Augusta, GA 30912, USA; qiwang@augusta.edu

**Keywords:** Alzheimer’s disease, exosomes, periodontitis

## Abstract

*Porphyromonas gingivalis* (Pg) and its gingipain proteases contribute to Alzheimer’s disease (AD) pathogenesis through yet unclear mechanisms. Cellular secretion of small extracellular vesicles or exosomes (EXO) increases with aging as part of the senescence-associated secretory phenotype (SASP). We have shown that EXO isolated from Pg-infected dendritic cells contain gingipains and other Pg antigens and transmit senescence to bystander gingival cells, inducing alveolar bone loss in mice in vivo. Here, EXO were isolated from the gingiva of mice and humans with/without periodontitis (PD) to determine their ability to penetrate the blood–brain barrier (BBB) in vitro and in vivo. PD was induced by Pg oral gavage for 6 weeks in C57B6 mice. EXO isolated from the gingiva or brain of donor Pg-infected (PD EXO) or control animals (Con EXO) were characterized by NTA, Western blot, and TEM. Gingival PD EXO or Con EXO were labeled and injected into the gingiva of uninfected WT mouse model. EXO biodistribution in brains was tracked by an in vivo imaging system (IVIS) and confocal microscopy. The effect of human PD EXO on BBB integrity and permeability was examined using TEER and FITC dextran assays in a human in vitro 3D model of the BBB. Pg antigens (RGP and Mfa-1) were detected in EXO derived from gingival and brain tissues of donor Pg-infected mice. Orally injected PD EXO from donor mice penetrated the brains of recipient uninfected mice and colocalized with hippocampal microglial cells. IL-1β and IL-6 were expressed in human PD EXO and not in Con EXO. Human PD EXO promoted BBB permeability and penetrated the BBB in vitro. This is the first demonstration that microbial-induced EXO in the oral cavity can disseminate, cross the BBB, and may contribute to AD pathogenesis.

## 1. Introduction

Alzheimer’s disease (AD) is currently the sixth leading cause of death in the US, but the exact mechanism of its pathogenesis remains unknown [1]. Periodontitis (PD) is a chronic inflammatory degenerative bone disease affecting more than 50% of the adult US population, with oral infectious etiology and increased prevalence in advanced age [2]. A role for periodontitis (PD) in the pathogenesis of AD has recently been proposed by several studies [3,4,5,6,7,8,9,10,11,12,13]. In addition, gingipains, proteolytic enzymes of the keystone oral pathogen *Porphyromonas gingivalis* (Pg), have been detected in the brains of AD patients postmortem [6]. A major knowledge gap exists in how the Gram-negative anaerobic coccobacillus Pg, invades the brain and how it may impact overall brain health.

Cellular senescence (CS), a stable cell cycle arrest, has been identified in several age-related diseases [14]. Chronic exposure to cell stressors can also induce premature senescence in the young [15]. A growing body of evidence in human and animal studies supports a role of cellular senescence in neurodegeneration and memory loss in AD [16]. Cellular senescence is known to increase biogenesis and secretion of exosomes (EXO), nano-sized membrane-enclosed particles, as part of the senescence-associated secretory pathway (SASP) [17]. EXO originate in the cellular endocytic pathway and are secreted in the extracellular space. It is worth noting that the blood–brain barrier (BBB) is highly vulnerable to penetration by EXO [18]. In addition, EXO derived from senescent cells can, in turn, transport molecular cargo that may induce senescence in bystander cells.

Our work has shown that Pg infection-induced PD stimulates senescence in gingival tissues and alveolar bone [19]. Dendritic cells (DC), a highly migratory immune cell that resides in the oral mucosal barrier, are vulnerable to Pg infection and senescence induction. This activates the SASP, consisting of inflammasome-related cytokines and EXO. The resulting EXO contain Pg antigens including gingipains and fimbriae adhesin proteins, which locally transmit and amplify immune senescence to bystander immune cells [17,19]. However, the capability of these pathogenic EXO to migrate systemically from gingiva to cross BBB and penetrate brain tissue has not been investigated yet.

The first aim of this study is to test the hypothesis that PD-induced EXO (PD-EXO) cross the BBB and deliver Pg virulence factors to brain tissue in vivo in mice. The second is to test the hypothesis that human PD-EXO cross BBB and increase brain microvascular endothelial cells’ (BMECs) permeability using an in vitro 3D model of human BBB.

## 2. Results

**Figure 1:**
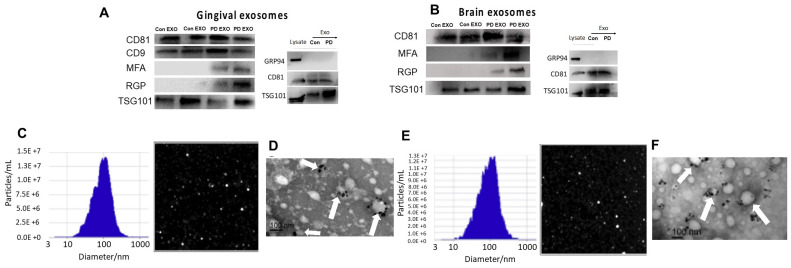
**Characterization of exosomes isolated from gingival and brain tissues of control and PD mice:** WB of control or PD mice EXO isolated from (**A**) gingival or (**B**) brain tissue showing positive markers (CD81, CD9, TSG101) and negative markers (GRP94) of EXO and Pg virulence factors, RGP, and Mfa1. Tissue lysate was used as a control. NTA analysis showing size (~100 nm) and concentration of EXO isolated from (**C**) gingival and (**E**) brain tissue. TEM showing EXO-positive marker CD63 with immunogold plating of EXO (white arrows) isolated from (**D**) gingival and (**F**) brain tissue, scale bar (100 nm).

**Exosomes isolated from gingiva and brain of PD mice express Pg gingipains (RGP) and Mfa-1.** Our study design for Pg-induced experimental PD in mice (PD mice) has been previously published [19,20]. EXO were isolated from gingival and brain tissues of control and PD mice. Characterization of PD mice exosomes (PD EXO) or control mice exosomes (Con EXO) was performed according to MISEV [21] using WB, NTA, and TEM. Analysis revealed the correct size, shape, and distinct positive markers (CD81, CD9, TSG101, CD63) and negative markers (GRP94) of EXO (Figure 1). Interestingly, virulence factors from Pg (RGP and Mfa-1) were detected in both gingival and brain tissue EXO of PD mice (Figure 1A,B). Phenotypically EXO from the gingiva and brain were identical, suggesting translocation of endogenously induced EXO from the gingiva to the brain.

**Figure 2:**
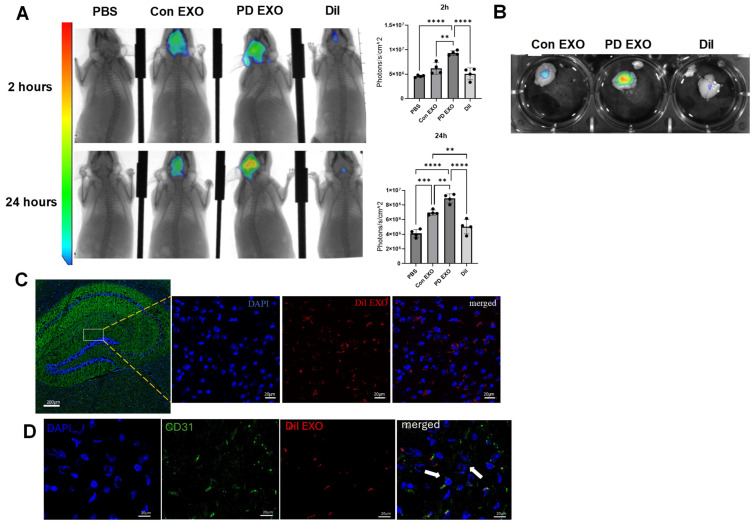
**PD EXO injected into the gingival tissues cross the BBB.** We injected 10^8^ particles of Dil labeled PD EXO or Con EXO into the gingival tissues of mice. (**A**) IVIS images of live mice and representative bar graphs 2 h (upper panel) and 24 h (lower panel). (**B**) Ex vivo IVIS images of brains harvested after euthanasia. Representative confocal microscopy showing (**C**) PD EXO (red) in the hippocampus of mice injected with PD EXO, nuclei in DAPI (blue), and (**D**) PD EXO (red), nuclei in DAPI (blue), CD31 (green) and merged showing colocalization (White arrows), scale bar (20 μm). Analysis was performed using one-way ANOVA and Tukey multiple comparison post hoc test (data are expressed as means ± SD, ** *p* < 0.01, *** *p* < 0.001, **** *p* < 0.0001).

**PD EXO translocate from gingival tissues to cross the blood–brain barrier in vivo**. To further test the ability of PD EXO to translocate to the brain tissue, EXO secreted from gingival tissues of PD or control mice were purified, fluorescently labeled, and injected intragingivally into the recipient WT mouse model (aged 6 months) as we described [19,20]. The purity of PD EXO (~99%) and lack of significant Pg outer membrane vesicles (OMV) were confirmed as previously described [19]. Biodistribution of fluorescent pre-labeled PD EXO or Con EXO was tracked at 2 and 24 h post-injection by in vivo live animal imaging system (IVIS) (Figure 2A) and ex vivo imaging of brains (Figure 2B). We observed a significantly higher signal in mice injected with PD EXO after 2 h compared to controls injected with Con EXO (Figure 2B). These findings suggest that the Pg antigen content of EXO may be responsible for compromising BBB integrity or its barrier function.

**Figure 3:**
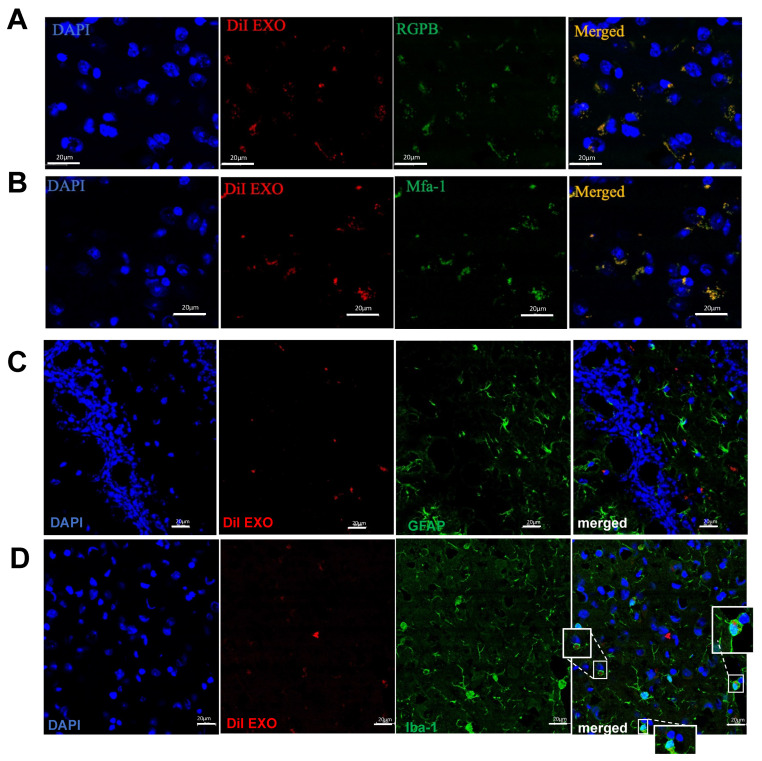
**PD EXO transport Pg virulence factors (RGP and Mfa1) to mice hippocampus and colocalize with microglial cells.** Representative confocal microscopy images showing the colocalization of DiI-labeled PD EXO (red) with: (**A**) Pg arginine-specific gingipains RGP (green) and (**B**) Pg Mfa-1 (green). Representative confocal microscopy images showing the colocalization (white box) of DiI-labeled PD EXO (red) with: (**C**) GFAP positive astrocytes (green) and (**D**) Iba1 positive microglial cell (green), scale bar (20 μm).

**PD EXO colocalize with microglia cells and deliver Pg antigens into the hippocampus of mice.** Detection of EXO was confirmed by confocal microscopy in the hippocampus of mice injected by PD EXO (Figure 2C), which were shown colocalized with endothelial cells (Figure 2D), Pg antigens RGP (Figure 3A), and Mfa1 (Figure 3B). Confocal microscopy revealed PD EXO colocalization with microglial cells (Figure 3D) and not astrocytes (Figure 3C).

**Figure 4:**
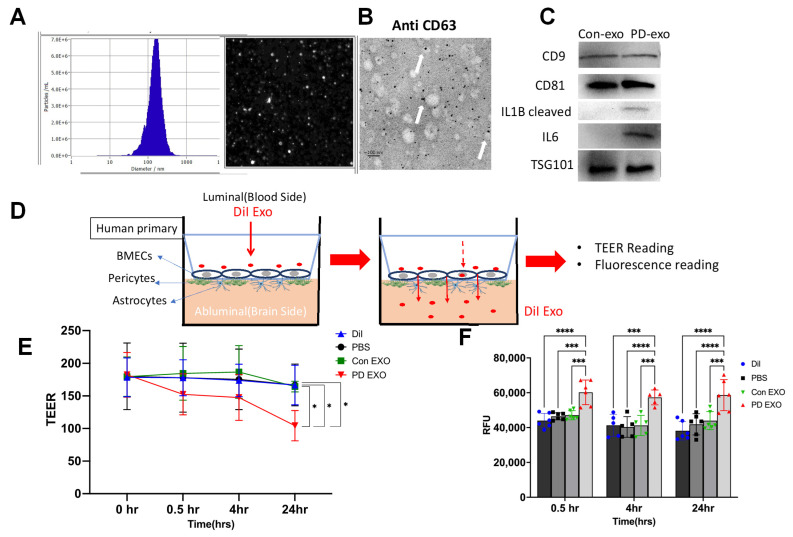
**Human PD EXO cross the BBB and compromise its barrier function.** Gingival tissues from healthy controls (Con) and PD patients were digested and EXO were isolated using ultracentrifugation. (**A**) NTA analysis showing the size of gingival EXO (~100 nm). (**B**) TEM showing immunogold labeling for CD63 (White arrows), scale bar (100 nm). (**C**) Representative WB showing markers of EXO isolated from gingival tissues of PD and Con patients. (**D**) Schematic diagram of in vitro 3D model of the BBB. (**E**) TEER readings of the BBB using an EVOM2 voltmeter at 0, 0.5, 4, and 24 h post addition of DiI-labeled EXO isolated from human gingival tissues of Con and PD patients or vehicle (PBS) or DiI only to the luminal side of the brain. (**F**) Fluorescent intensity of the media from the abluminal side of the BBB was obtained by a plate reader at 0.5, 4, and 24 h post addition of DiI EXO. Analysis was performed using two-way ANOVA and Dunn’s correction test. (Data are expressed as means ± SD, * *p* < 0.05, *** *p* < 0.001, **** *p* < 0.0001).

**Human periodontitis-induced EXO (PD EXO) cross the BBB and induce barrier dysfunction.** To further validate our data on mice PD EXO, EXO were purified from human gingiva of n = 7 stage III–IV, grade A/B PD [22] (PD-EXO), and healthy subjects (n = 5). The correct size range (30–150 nm) was assessed by NTA (Figure 4A). EXO shape and expression of the exosomal marker were confirmed by SEM (Figure 4B). Immunoblot analysis revealed expression of IL-1β and IL-6 in EXO isolated from gingival tissues of PD patients while not being detected in healthy controls (Figure 4C). To test if PD-triggered EXO from gingival tissue can cross BBB, we used a human BBB in vitro 3D model (Figure 4D). DiI pre-labeled PD EXO were added to the luminal side (endothelial cells) of an in vitro 3D model of the BBB after baseline (0 h) and TEER measurement reached more than 150 Ω. PD EXO significantly decreased TEER measurements at 0.5, 4, and 24 h post-treatment relative to EXO from healthy subjects (Con EXO) (Figure 4E). Moreover, PD EXO could cross the BBB, as shown by an increased DiI fluorescent signal in the lower compartment (abluminal side) of the BBB compared to Con EXO and vehicle (PBS) (Figure 4F). The DiI label itself was ineffective at crossing the BBB, showing that PD-induced EXO could cross the BBB and compromise its integrity.

**Figure 5:**
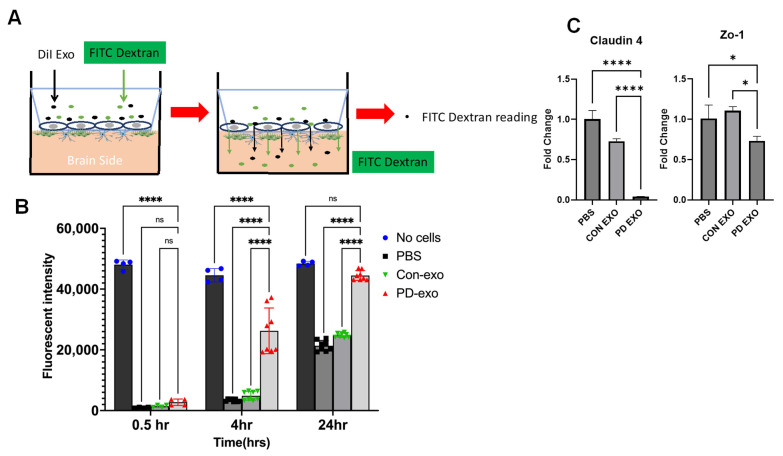
**In vitro permeability assay in a human 3D model of the BBB.** (**A**) Schematic diagram of the permeability assay in vitro 3D model of BBB. (**B**) Fluorescent intensity of the media from the abluminal side of the BBB obtained by a plate reader at 0.5, 4, and 24 h post addition of FITC dextran and of EXO isolated from human gingival tissues of healthy and PD patients or PBS. No cells group was used as a control. Analysis was performed using two-way ANOVA and Dunn’s correction test. (**C**) mRNA expression of tight junction proteins Claudin4 and ZO-1 (data are expressed as means ± SD, * *p* < 0.05, **** *p* < 0.0001).

**Human periodontitis-induced EXO (PD EXO) increase BBB permeability.** To test the effect of EXO released from gingival tissues of PD patients (PD EXO) on BBB permeability, we added FITC dextran to the luminal side of the BBB simultaneously with EXO addition and sampled the media in the abluminal side for fluorescent measurement with a plate reader at 0.5, 4, and 24 h post-treatment (Figure 5A). Figure 5B shows a significant increase in the FITC fluorescent signal with PD EXO at 4 h and 24 h while there was an equal slight increase in the signal with PBS and EXO from healthy gingiva at the 24 h time point and a constitutive high signal in the “no cells” group at all time points, showing the role of PD EXO in increasing BBB permeability. Furthermore, there was a significant downregulation of tight junction mRNA gene expression claudin 4 and Zo-1 in the BBB brain microvascular endothelial cells (BMECs) (Figure 5C).

## 3. Discussion

Alzheimer’s disease and related dementia (ADRD) affect 50 million people worldwide with no effective cure. Currently, more than 6.2 million Americans aged 65 years and older are suffering from AD, with a projection of that number doubling in 2060 [1]. A role for infectious disease in the pathogenesis of AD has long been proposed [23] with viruses such as HSV [24] and, more recently, gingipains of the oral pathogen *Porphyromonas gingivalis* (Pg) [6] being detected in the brains of AD patients postmortem. There is a strong epidemiologic association between periodontitis (PD) and ADRD [5,6,7,8,9,10,11,12,13] but how these two diseases intersect and whether it is at the level of causation is unclear. Recent evidence shows a role for Pg and its potent proteases gingipains in AD pathology [6]; however, definitive evidence for the presence of Pg whole bacterium in the brain tissues is lacking and the precise mechanism of microbial invasion of the BBB is not clearly understood. Here we show that PD EXO from host cells in the inflamed oral mucosa could act as a long-distance carrier for delivery and release of gingipains and other virulence factors to local and distant sites such as brain tissue.

The extracellular microenvironment directs the activity and genetic, molecular, and functional profile of the cells. EXO are an integral component of the extracellular milieu. EXO can cross the BBB and have been shown to have a role in the transfer of genetic information from peripheral hematopoietic cells and to induce neuroinflammation in the brain [18]. Pg-induced EXO of the SASP express integrins and lectins that promote cell binding at the inflamed site [19,20]. Moreover, those EXO are enriched with virulence factors from Pg including gingipains, a potent proteolytic enzyme, enabling them to disrupt barrier functions of the epithelium and penetrate through interstitial spaces and lymphatics to find their way into systemic circulation. Compared to free molecules, EXO act as a “Trojan Horse”, bringing pathogenic cargo enclosed in a stable lipid membrane for delivery to target tissues. Here we show for the first time that EXO purified from both gingival and brain tissue of PD mice express Pg gingipains (RGP) and Mfa-1. Additionally, orally administrated PD EXO were capable of penetrating the BBB in mice. These data suggest that Pg infection induces senescence in gingival tissues of PD mice [19] and increases the release of pathogenic EXO that carry pathogen-related and SASP molecules to distant bystander brain cells.

Cellular senescence [25] plays a crucial role in AD pathogenesis. Our group showed previously that PD EXO could transmit senescence to gingival immune cells, including DCs and Tells [17,19]. Microglial cells are resident housekeeping phagocytes of the central nervous system that have a crucial function in injury response, immune defense, and synaptic remodeling [26]. In addition, several studies showed the paramount role of microglial cells in maintaining tissue homeostasis and keeping the extracellular space clean of β-amyloid (Aβ), thereby preventing AD [27]. The co-localization of PD EXO with hippocampus microglial cells and not astrocytes highlighted the target cells of PD EXO in mouse brain tissue. Moreover, removal of senescent microglial cells was shown to inhibit neuroinflammation and cognitive impairment [28]. This suggests the potentiality of microglial cells as a therapeutic cellular target for oral microbially-induced AD.

The breakdown of the blood–brain barrier (BBB) plays a critical role in the progression of AD [29]. This disruption in BBB integrity is caused by the downregulation or derangement of brain microvascular endothelial cells (BMECs) tight junction proteins. Gingipains are potent proteolytic enzymes produced by *Pg* that degrade tight junction proteins of human endothelial cells [3,4]. In addition, gingipains were detected in brain tissue samples of AD patients [6]. Our data show that EXO from gingival and brain tissues of PD mice contain gingipains. In addition, human PD EXO crossed the human 3D in vitro model of the BBB (Figure 4). EXO were found to utilize two primary routes to traverse the BBB: paracellular, which involves tight and adherens junctions, and transcellular, which involves micropinocytosis, receptor, or caveola-mediated endocytosis [30]. The human 3D in vitro model of the BBB used in this study showed that EXO released in human gingival tissues during PD promotes BMEC dysfunction by degrading tight junction proteins, increasing BBB permeability.

Inhibition of PD EXO release can be achieved by local intraoral injection of senomorphic agent rapamycin [19,31] or GW4869 [32], a neutral sphingomyelinase inhibitor. In addition, modulating local immune responses in periodontitis lesions with tolerogenic DCS and T regulatory cells could inhibit SASP EXO release [19,20]. Although the current study aims to determine the link between PD and neurogenerative diseases including AD, the lack of an AD mice model is a limitation. In addition, the human BBB used is an in vitro model that may not reflect the same physiological response in the in vivo settings.

In conclusion, this study provides a new insight into the context of neurodegenerative diseases by identifying EXO as a new biological mechanism for crosstalk between microbial pathogens and brain tissue. Oral microbially-induced EXO (PD-EXO) can pass through the blood–brain barrier and can traffic inflammatory molecules and bacterial virulence factors to brain cells. Our group is currently studying the molecular mechanism underlying the interaction of PD EXO with the BBB and targeted senolytic therapies for periodontitis and AD.

## 4. Methods and Materials

### 4.1. Experimental PD in Mice Model

The Institutional Animal Care and Use Committee (IACUC) of Augusta University (protocol #2022-1073) approved all experimental procedures. The PD model has been published by our group previously [19,20]. In this study, PD was induced in (4–5 mo) male C57BL6 mice. Control Group: received only 2% CMC vehicle by oral gavage. PD Group: received Pg in 2% CMC by oral gavage. Oral gavages were carried out every other day for a total of six weeks [6] to induce PD in mice. *P. gingivalis* 381 (Pg) was maintained anaerobically in (10%, H2, 10% CO2, and 80% N2) in a Coy lab vinyl anaerobic chamber (Coy Laboratory Products, Inc., Grass Lake, MI, USA) at 37 °C in Wilkins–Chalgren anaerobe broth. Bacterial cells were maintained until the mid-log phase. Bacterial colony forming units (CFU) were calculated based on a spectrophotometer OD 660 reading of 0.11, previously reported to equal 5 × 10^7^ CFU.10^9^ CFU of Pg, were suspended in 2%CMC (Carboxymethyl cellulose) in sterile PBS and administered to animals via oral gavage. At the end of the 6 weeks, animals were euthanized and brain and gingival tissues were harvested from the same animals and further processed for Western blotting and confocal microscopy.

### 4.2. Periodontitis Patients and Healthy Volunteers

Human gingival tissues from healthy controls (Con) (mean age 45 years) and PD patients (mean age 51 years) (n = 4) stage III–IV, grade B/C PD were obtained under institutional review board (IRB)-approved protocols (IRB NET ID: 1169935-21). All subjects provided informed consent prior to participation in the study.

### 4.3. Brain Exosome Isolation

The whole brain was dissected from the mouse and approximately 0.06 g of brain tissue was excised from the forebrain and placed in PBS on ice. The tissue was transferred to 2 mL of RPMI media and then cut into approximately 2 mm pieces. To digest the tissue, Collagenase D was added at a final concentration of 2 mg/mL and DNase I was added at a final concentration of 40 U/mL (Sigma Aldrich, Burlington, MS, USA). Subsequently, tissues and media were incubated at 37 °C with shaking at 75 RPM for 30 min. In order to isolate exosomes from the samples, tissues and media were passed through a 70 µm strainer and gently mushed with a sterile syringe plunger to avoid cell dissociation. The strainer was then thoroughly rinsed with 1 mL of RPMI media. To stop enzyme activity, protease and phosphatase inhibitor was added at a final concentration of 1× (Thermofisher Scientific, Waltham, MA, USA). Afterward, the supernatant was successively centrifugated at 300× *g* for 10 min, 2000× *g* for 20 min, and 4000× *g* for 20 min to remove debris and cells. Afterward, ultrafiltration 1 time with a 0.45 µm filter, 2× with 100 kDa filters, and ultracentrifugation for 1.5 h at 120,000× *g* were performed. The EXO pellets were resuspended in 100 uL of PBS and stored at −80 °C for further studies.

### 4.4. Gingival Exosome Isolation

Mice or human gingival tissues were isolated and dissected and placed in PBS on ice. The tissues were pooled together for each group and were cut into quarters in RPMI media with Collagenase D added at a final concentration of 2 mg/mL and DNase I at a final concentration of 40 U/mL (Sigma Aldrich, Burlington, MS, USA) and then incubated at 37 °C for 30 min. Afterward, tissues and media were gently mushed through a 70 µm strainer on top of a petri dish. The plate and strainer were rinsed thoroughly and then protease/phosphatase inhibitor was added to prevent further enzyme activity (Thermofisher Scientific, Waltham, MA, USA; cat#: 78443). The supernatants were subjected to subsequent centrifugation at 300× *g* for 10 min, 2000× *g* for 20 min, and 4000× *g* for 20 min to eliminate all cells and debris, followed by ultrafiltration with a 0.45 µm filter and 2× with 100 kDa filters. To remove additional free proteins, samples were ultra-centrifugated for 1.5 h at 120,000× *g*. The EXO pellets were then resuspended in 100 uL of PBS and stored at −80 °C for further studies [19].

### 4.5. Nanoparticle Tracking Analysis of Exosomes

Nanoparticle tracking analysis (NTA) was used to analyze and visualize the size and count of EXO in suspension [19,20,33]. Briefly, 10 µL of the sample was diluted to a final volume of 1 mL using 1× PBS buffer and was loaded into the sample chamber of the ZetaView PMX 120 instrument (Particle Metrix, Meerbusch, Germany) at 23 °C. Data about the size distribution and concentration of the sample were generated by the ZetaView software (8.02.28).

### 4.6. Electron Microscopy

As we described previously [17], the EXO sample was fixed in 4% paraformaldehyde in 0.1M cacodylate buffer PH 7.4 overnight. Five microliters (5 µL) of suspended EXO preparation were applied to a carbon-Formvar coated 200 mesh nickel grid and allowed to stand for 30 min. The excess sample was wicked off onto Whatman filter paper. Grids were floated EXO side down onto a 20 µL drop of 1M Ammonium Chloride for 30 min to quench aldehyde groups from the fixation step. Grids were floated on drops of blocking buffer (0.4% BSA in PBS) for 2 h, then rinsed 3× with PBS (5 min each). Grids were set up as follows and allowed to incubate in blocking buffer or the primary antibody anti-CD63 (#PA5-92370) (Thermofisher Scientific, Waltham, MA, USA) for 1 h. Grids were floated on drops of 1.4 nm secondary antibody nanogold (Nanoprobes, Inc., Yaphank, NY, USA) diluted 1:1000 in blocking buffer for 1 h. Grids were rinsed 3× for 5 min each with DI. For visibility in the electron SEM, the exosome sample was postfixed in 2% osmium tetroxide in NaCac buffer and dehydrated in ethanol. Then, the sample was mounted on aluminum stubs and sputter-coated for 6 min with gold–palladium (Anatech Hummer 6.2, Union City, CA, USA). EXO were observed and imaged at 10 kV using an FEI XL30 scanning electron microscope (FEI, Hillsboro, OR, USA).

### 4.7. Western Blotting and Antibodies

As previously described [17], gingival or brain EXO or cell lysates were extracted by the addition RIPA buffer with a protease/phosphatase inhibitor cocktail and incubation was undertaken for 30 min on ice. After denaturation, protein separation was performed utilizing 4–15% Mini-PROTEAN TGX Precast Protein Gel (Bio-Rad Laboratories, Hercules, CA, USA; Cat#: 4568084), then transferred to PVDF membranes (Bio-Rad laboratories, Hercules, CA, USA; Cat#: 1620177). Subsequently, membranes were blocked with 5% nonfat dry milk in TBST for 1 h followed by incubation with primary antibodies at 4◦ overnight. After washing with TBST, membranes were incubated with HRP-conjugated secondary antibodies for 1 h at room temperature the following day. Membranes were washed and developed with a Femto kit (Thermofisher Scientific, Waltham, MA, USA; Cat#: 34095) and imaged with ChemiDoc MP Imaging Gel (Bio-Rad laboratories, Hercules, CA, USA). Antibodies used were anti-mouse/anti-human TSG101 (MA1-23296) (Thermofisher Scientific, Waltham, MA, USA), anti-human CD81 (#10037), anti-human/anti-mouse CD9 (#13174) (Thermofisher Scientific, Waltham, MA, USA), anti-mouse IL-6 (#12912S) (Cell Signaling Technology, Danvers, MA, USA), anti-human IL-6 (#12153) (Cell Signaling Technology, Danvers, MA, USA), anti-human IL-1B (#12703) (Cell Signaling Technology, Danvers, MA, USA), anti-mouse IL1B (#12426) (Cell Signaling Technology, Danvers, MA, USA), anti-Grp94 (#2104) (Cell Signaling Technology, Danvers, MA, USA), anti-CD81 (mouse-specific) (#10037) (Cell Signaling Technology, Danvers, MA, USA), anti-Mfa1 (generated at the Cell Culture/Hybridoma Facility at Stony Brook University, as reported [34,35]), and anti Rgp antibody (provided as courtesy from Dr Jan Potempa, as reported [6]). Secondary antibodies used were anti-mouse IgG HRP-linked (#7076) (Cell Signaling Technology, Danvers, MA, USA) and anti-rabbit IgG HRP-linked (#7074) (Cell Signaling Technology, Danvers, MA, USA) antibodies.

### 4.8. Biodistribution of Gingival EXO after Intragingival Injection

To assess the ability of gingival PD EXO to cross the BBB, EXO were Dii-labeled and tracked in vivo, as previously described [19,20]. Briefly, 10^8^ control or PD gingival EXO were labeled with Vybrant™ DiI Cell-Labeling Solution (Thermofisher) and injected in the mice palatal gingiva mice (n = 6 mice/group). Biodistribution of injected DiI-labeled EXO was quantitated by an in vivo imaging system (xenogen IVIS Lumina) at 2 and 24 h. Ex vivo fluorescence measurements of the harvested brain were performed to confirm the in vivo EXO tracking.

### 4.9. Gingival EXO Colocalization with Pg Mfa-1, Pg Gingipains, Microglial Cells, Astrocytes, and Endothelial Cells in Brain In Vivo

Cryosections of brain tissues that had been excised and frozen were mounted on slides and stored at −80 °C for further processing. Frozen brain tissue sections were allowed to thaw at room temperature and then rinsed with PBS (no Ca, no Mg). Tissue sections were then fixed with 4% paraformaldehyde (PFA) for 10 min at room temperature followed by washing 3× in PBS to remove the fixative solution. For labeling of Pg minor fimbria (Mfa-1), Pg arginine specific gingipains (Rgp), microglial cells, endothelial cells, and astrocytes, tissues were permeabilized with 0.1% Triton x-100 for 10 min followed by rinsing 3× in PBS. Then, slides were incubated overnight at 4 °C with anti-Iba-1 antibody (Invitrogen, Waltham, MA USA, Cat#PA5-27436), anti-GFAP antibody (Invitrogen, Waltham, MA USA; Cat#PA1-10019), anti-CD31 antibody (Invitrogen, Waltham, MA USA; Cat#MA5-37858), anti-Rgp (provided as courtesy from Dr Jan Potempa, as reported [6]), and anti-Mfa-1 (generated at the Cell Culture/Hybridoma Facility at Stony Brook University, as reported [21,35]), at a dilution of 1:1000. The next day, slides were washed 3× with PBS and then incubated with a secondary antibody; Goat anti-Mouse IgG, DyLight 488 (R&D Systems; Minneapolis, MN, USA, Goat anti-Rabbit IgG (H + L), Alexa Fluor 488 (Invitrogen, Waltham, MA USA; Cat#-32731) for 1 h at room temperature with subsequent washing 3× with PBS (5 min each). Then, slides were mounted with DAPI, and images were captured with a Zeiss 780 upright confocal microscope (Carl Zeiss, AG, Oberkochen, Germany).

### 4.10. Human In Vitro Blood–Brain Barrier Model Culture and PD EXO Treatment

A 3D Human Blood Brain Barrier (BBB) kit was cultured and incubated following the manufacturer’s instructions at 37 °C with 5% CO2 (Neuromics, Edina, MN, USA; Cat#3D45002). The blood–brain barrier (BBB) model consists of co-cultures of human brain endothelial cells, human brain pericytes, and human brain astrocytes and was stored at −80 °C until they were ready for use. Briefly, cells were allowed to thaw then freezing media was replaced with medium 1 (Blood–Brain Barrier Growth Media). Once completely thawed, fresh medium 1 was added to the model and incubated at 37 °C with 5% CO2 for 2.5 h. Subsequently, medium 1 was removed and replaced with medium 2 (Endo-Neuro-Pharmaceuticals Media), and the BBB kit was replaced back in the incubator until Day 4 after thawing. On days 4, 5, and 6, medium 2 was replaced with medium 3, and trans-endothelial electrical resistance (TEER) readings were acquired to determine cell activation. Model TEER readings reaching 150 Ω × cm^2^ or greater were considered sufficient for further studies. TEER readings were performed by an EVOM^2^ Epithelial Voltohmeter (World Precision Instruments, Inc., Sarasota, FL, USA; Cat#: 013013). Once cells were activated (>150 Ω × cm^2^), DiI-labeled gingival human control or PD exosomes were added to demonstrate the permeability of the BBB by exosomes. Exosomes labeled with DiI (Invitrogen, Thermofisher Scientific, Waltham MA, USA; Cat#: C7001) were added to the top of the insert and incubated for 24 h at 37 °C with 5% CO2. At the time points of 30 min, 4 h, and 24 h, TEER readings were recorded, and 100 uL of the media in the lower chamber was collected for quantification. The fluorescence intensity of the collected media was measured using Biotek Synergy H1 microplate reader (Biotek, Winooski, VT, USA Cat#: 8041005) and corresponding software Gen 5 3.09 at an absorbance of 540 and an emission of 575. Groups were as follows: PBS, gingival human Con, and PD EXO and DiI only.

### 4.11. FITC-Dextran Permeability Assay in Blood Brain Barrier Model

Once cells were activated (>150 Ω × cm^2^), FITC-dextran (1 mg/mL) was added to the insert to demonstrate the permeability of the BBB. EXO were also added to the top of inserts and incubated for 24 h at 37 °C with 5% CO2. Groups were: PBS, gingival human Con and PD EXO, and a group where no cells were added. At the time points of 30 min, 4 h, and 24 h, TEER readings were recorded, and 100 uL of the media in the lower chamber was collected for quantification. The fluorescence intensity of the collected media was measured using Biotek Synergy H1 microplate reader (Biotek, Winooski, VT, USA Cat#: 8041005) and corresponding software Gen 5 3.09 at an absorbance of 510 and emission of 550.

#### Real-Time PCR

Total RNA was isolated from human BBB brain microvascular endothelial cells (BMECs) using a QIAGEN RNeasy mini kit (Qiagen, Inc., Valencia, CA, USA). RNA purity and concentration were measured using Nanodrop (NanoDrop 1000 UV-VIS Spectrophotometer Software Ver.3.8.1, Thermofisher Scientific). A ratio of 260/280 of 2.0 was considered adequate for analysis. Reverse transcription to cDNA was performed using the High-Capacity cDNA Reverse Transcription Kit (Applied Biosystem, Thermofisher Scientific, Waltham MA, USA) in a total reaction of 20 μL. Quantitative real-time PCR was performed using Taqman fast advanced master mix (Applied Biosystem, Thermofisher Scientific, Waltham MA, USA) and TaqMan Gene Expression assay (Applied Biosystem, Foster City, CA, USA) specific for: Claudin 4 (Hs00976831_s1), ZO-1 (Hs01551871_m1), and internal control beta actin (Actb) (Hs01060665_g1). RT-PCR was run using the StepOnePlus Real-Time PCR System. Calculation of relative mRNA expression was performed using delta–delta CT and presented as relative fold-change to the control group.

### 4.12. Statistical Analysis

Data were analyzed using GraphPad Prism 10 (GraphPad Software, La Jolla, CA, USA). One-way or two-way ANOVA with significance defined as *p* < 0.05 and a confidence level of 95% confidence interval followed by Tukey’s multiple-comparisons test were used for data analysis. Values are expressed as mean ± standard deviation (SD) and experiments were repeated 3 times.

## Data Availability

The raw data supporting the findings of this study will be made available by the authors upon reasonable request.

## References

[1] Lane C.A., Hardy J., Schott J.M. (2021). 2021 Alzheimer’s disease facts and figures. Alzheimer’s Dement..

[2] Eke P.I., Thornton-Evans G., Dye B., Genco R. (2012). Advances in Surveillance of Periodontitis: The Centers for Disease Control and Prevention Periodontal Disease Surveillance Project. J. Periodontol..

[3] Nonaka S., Kadowaki T., Nakanishi H. (2022). Secreted gingipains from *Porphyromonas gingivalis* increase permeability in human cerebral microvascular endothelial cells through intracellular degradation of tight junction proteins. Neurochem. Int..

[4] Zou Z., Fang J., Ma W., Guo J., Shan Z., Ma D., Hu Q., Wen L., Wang Z. (2023). *Porphyromonas gingivalis* Gingipains Destroy the Vascular Barrier and Reduce CD99 and CD99L2 Expression To Regulate Transendothelial Migration. Microbiol. Spectr..

[5] Jungbauer G., Stähli A., Zhu X., Alberi L.A., Sculean A., Eick S. (2022). Periodontal microorganisms and Alzheimer disease—A causative relationship?. Periodontology 2000.

[6] Dominy S.S., Lynch C., Ermini F., Benedyk M., Marczyk A., Konradi A., Nguyen M., Haditsch U., Raha D., Griffin C. (2019). *Porphyromonas gingivalis* in Alzheimer’s disease brains: Evidence for disease causation and treatment with small-molecule inhibitors. Sci. Adv..

[7] Nara P.L., Sindelar D., Penn M.S., Potempa J., Griffin W.S.T. (2021). *Porphyromonas gingivalis* Outer Membrane Vesicles as the Major Driver of and Explanation for Neuropathogenesis, the Cholinergic Hypothesis, Iron Dyshomeostasis, and Salivary Lactoferrin in Alzheimer’s Disease. J. Alzheimer’s Dis..

[8] Ryder M.I., Xenoudi P. (2021). Alzheimer disease and the periodontal patient: New insights, connections, and therapies. Periodontology 2000.

[9] Gong T., Chen Q., Mao H., Zhang Y., Ren H., Xu M., Chen H., Yang D. (2022). Outer membrane vesicles of *Porphyromonas gingivalis* trigger NLRP3 inflammasome and induce neuroinflammation, tau phosphorylation, and memory dysfunction in mice. Front. Cell. Infect. Microbiol..

[10] Fan Z., Tang P., Li C., Yang Q., Xu Y., Su C., Li L. (2023). *Fusobacterium nucleatum* and its associated systemic diseases: Epidemiologic studies and possible mechanisms. J. Oral Microbiol..

[11] Noble J.M., Borrell L.N., Papapanou P.N., Elkind M.S.V., Scarmeas N., Wright C.B. (2009). Periodontitis is associated with cognitive impairment among older adults: Analysis of NHANES-III. J. Neurol. Neurosurg. Psychiatry.

[12] Qi X., Zhu Z., Plassman B.L., Wu B. (2021). Dose-Response Meta-Analysis on Tooth Loss With the Risk of Cognitive Impairment and Dementia. J. Am. Med. Dir. Assoc..

[13] Nadim R., Tang J., Dilmohamed A., Yuan S., Wu C., Bakre A.T., Partridge M., Ni J., Copeland J.R., Anstey K.J. (2020). Influence of periodontal disease on risk of dementia: A systematic literature review and a meta-analysis. Eur. J. Epidemiol..

[14] Kumari R., Jat P. (2021). Mechanisms of Cellular Senescence: Cell Cycle Arrest and Senescence Associated Secretory Phenotype. Front. Cell Dev. Biol..

[15] Palacio L., Goyer M., Maggiorani D., Espinosa A., Villeneuve N., Bourbonnais S., Moquin-Beaudry G., Le O., Demaria M., Davalos A.R. (2019). Restored immune cell functions upon clearance of senescence in the irradiated splenic environment. Aging Cell.

[16] Liu R.-M. (2022). Aging, Cellular Senescence, and Alzheimer’s Disease. Int. J. Mol. Sci..

[17] Elsayed R., Elashiry M., Liu Y., El-Awady A., Hamrick M., Cutler C.W. (2021). *Porphyromonas gingivalis* Provokes Exosome Secretion and Paracrine Immune Senescence in Bystander Dendritic Cells. Front. Cell. Infect. Microbiol..

[18] Ridder K., Keller S., Dams M., Rupp A.-K., Schlaudraff J., Del Turco D., Starmann J., Macas J., Karpova D., Devraj K. (2014). Extracellular vesicle-mediated transfer of genetic information between the hematopoietic system and the brain in response to inflammation. PLOS Biol..

[19] Elsayed R., Elashiry M., Liu Y., Morandini A.C., El-Awady A., Elashiry M.M., Hamrick M., Cutler C.W. (2023). Microbially-Induced Exosomes from Dendritic Cells Promote Paracrine Immune Senescence: Novel Mechanism of Bone Degenerative Disease in Mice. Aging Dis..

[20] Elashiry M., Elashiry M.M., Elsayed R., Rajendran M., Auersvald C., Zeitoun R., Rashid M.H., Ara R., Meghil M.M., Liu Y. (2020). Dendritic cell derived exosomes loaded with immunoregulatory cargo reprogram local immune responses and inhibit degenerative bone disease in vivo. J. Extracell. Vesicles.

[21] Théry C., Witwer K.W., Aikawa E., Alcaraz M.J., Anderson J.D., Andriantsitohaina R., Antoniou A., Arab T., Archer F., Atkin-Smith G.K. (2018). Minimal information for studies of extracellular vesicles 2018 (MISEV2018): A position statement of the International Society for Extracellular Vesicles and update of the MISEV2014 guidelines. J. Extracell. Vesicles.

[22] Tonetti M.S., Greenwell H., Kornman K.S. (2018). Staging and grading of periodontitis: Framework and proposal of a new classification and case definition. J. Periodontol..

[23] Moir R.D., Lathe R., Tanzi R.E. (2018). The antimicrobial protection hypothesis of Alzheimer’s disease. Alzheimer’s Dement..

[24] Middleton P., Petric M., Kozak M., Rewcastle N., McLachlan D.C. (1980). Herpes-simplex viral genome and senile and presenile dementias of alzheimer and pick. Lancet.

[25] Guerrero A., De Strooper B., Arancibia-Cárcamo I.L. (2021). Cellular senescence at the crossroads of inflammation and Alzheimer’s disease. Trends Neurosci..

[26] Nayak D., Roth T.L., McGavern D.B. (2014). Microglia Development and Function. Annu. Rev. Immunol..

[27] Hansen D.V., Hanson J.E., Sheng M. (2018). Microglia in Alzheimer’s disease. J. Cell Biol..

[28] Ogrodnik M., Evans S.A., Fielder E., Victorelli S., Kruger P., Salmonowicz H., Weigand B.M., Patel A.D., Pirtskhalava T., Inman C.L. (2021). Whole-body senescent cell clearance alleviates age-related brain inflammation and cognitive impairment in mice. Aging Cell.

[29] Nation D.A., Sweeney M.D., Montagne A., Sagare A.P., D’Orazio L.M., Pachicano M., Sepehrband F., Nelson A.R., Buennagel D.P., Harrington M.G. (2019). Blood–brain barrier breakdown is an early biomarker of human cognitive dysfunction. Nat. Med..

[30] Chen C.C., Liu L., Ma F., Wong C.W., Guo X.E., Chacko J.V., Farhoodi H.P., Zhang S.X., Zimak J., Ségaliny A. (2016). Elucidation of Exosome Migration Across the Blood–Brain Barrier Model In Vitro. Cell. Mol. Bioeng..

[31] An J.Y., Quarles E.K., Mekvanich S., Kang A., Liu A., Santos D., Miller R.A., Rabinovitch P.S., Cox T.C., Kaeberlein M. (2017). Rapamycin treatment attenuates age-associated periodontitis in mice. GeroScience.

[32] Essandoh K., Yang L., Wang X., Huang W., Qin D., Hao J., Wang Y., Zingarelli B., Peng T., Fan G.-C. (2015). Blockade of exosome generation with GW4869 dampens the sepsis-induced inflammation and cardiac dysfunction. Biochim. Biophys. Acta.

[33] Helwa I., Cai J., Drewry M.D., Zimmerman A., Dinkins M.B., Khaled M.L., Seremwe M., Dismuke W.M., Bieberich E., Stamer W.D. (2017). A Comparative Study of Serum Exosome Isolation Using Differential Ultracentrifugation and Three Commercial Reagents. PLoS ONE.

[34] Carrion J., Scisci E., Miles B., Sabino G.J., Zeituni A.E., Gu Y., Bear A., Genco C.A., Brown D.L., Cutler C.W. (2012). Microbial carriage state of peripheral blood dendritic cells (DCs) in chronic periodontitis influences DC differentiation, atherogenic potential. J. Immunol..

[35] Zeituni A.E., McCaig W., Scisci E., Thanassi D.G., Cutler C.W. (2010). The Native 67-Kilodalton minor fimbria of *Porphyromonas gingivalis* is a novel glycoprotein with DC-SIGN-targeting motifs. J. Bacteriol..

